# [Corrigendum] Icaritin acts synergistically with epirubicin to suppress bladder cancer growth through inhibition of autophagy

**DOI:** 10.3892/or.2026.9056

**Published:** 2026-01-21

**Authors:** Xiu-Wu Pan, Lin Li, Yi Huang, Hai Huang, Dan-Feng Xu, Yi Gao, Lu Chen, Ji-Zhong Ren, Jian-Wei Cao, Yi Hong, Xin-Gang Cui

Oncol Rep 35: 334–342, 2016; DOI: 10.3892/or.2015.4335

Following the publication of the above article, the authors have contacted the Editorial Office to explain that they had noticed that, in Fig. 4 on p. 339, the same western blot data for the ATG5 protein had inadvertently been included for the T24 and the BT5637 cell lines for the 72 h experiments (the lower panels of blots).

However, the authors had retained their original data, and were able to identify how this error occurred. The revised version of Fig. 4, now showing the correct data for the ATG5 protein for the 72 h experiment with the BT5637 cell line, is shown on the next page. Note that this error did not affect the overall results and conclusions reported in the paper. The authors are grateful to the Editor of *Oncology Reports* for granting them the opportunity to publish this corrigendum, and all the authors agree with its publication; furthermore, they apologize to the readership of the journal for any inconvenience caused.

## Figures and Tables

**Figure 4.**
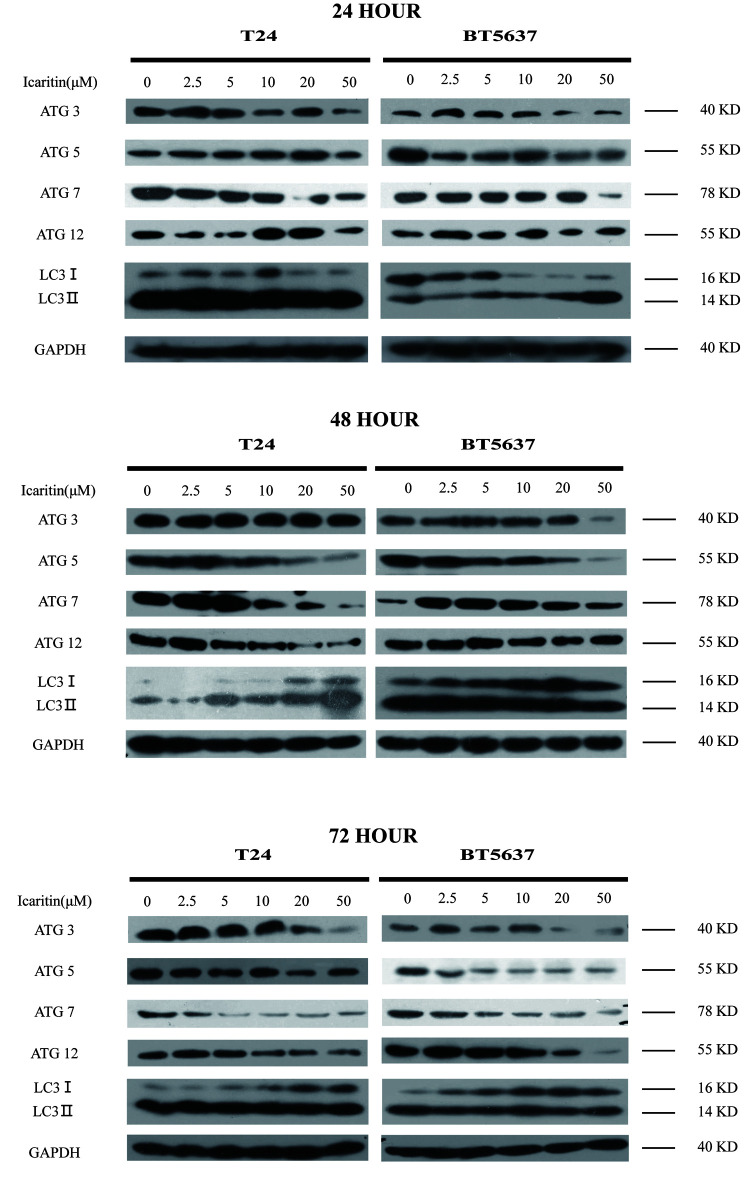
Icaritin induced autophagy suppression. Both bladder cancer cells T24 and BT5637 were treated with icaritin in 2.5, 5, 10, 20 and 50 µM for 24, 48 and 72 h. Proteins of ATG8 conjugation system including ATG3, ATG5, ATG7 and ATG12 were detected. Additionally, both autophagosome marker LC3-I and LC3-II were discovered.

